# Active Disturbance Rejection Control in Magnetic Bearing Rotor Systems with Redundant Structures

**DOI:** 10.3390/s22083012

**Published:** 2022-04-14

**Authors:** Baixin Cheng, Xin Cheng, Shao Song, Huachun Wu, Yefa Hu, Rougang Zhou, Shuai Deng

**Affiliations:** 1 School of Mechanical & Electronic Engineering, Wuhan University of Technology, Wuhan 430070, China; chengbaixin@126.com (B.C.); chengx@whut.edu.cn (X.C.); whc@whut.edu.cn (H.W.); huyefa@whut.edu.cn (Y.H.); dengshuai2014@163.com (S.D.); 2 Hubei Maglev Engineering Technology Research Center, Wuhan University of Technology, Wuhan 430070, China; 3 China Ship Development and Design Center, Wuhan 430070, China; songshao1986@126.com; 4 Shenzhen Research Institute, Wuhan University of Technology, Wuhan 430070, China; 5 School of Mechanical Engineering, Hangzhou Dianzi University, Hangzhou 310000, China

**Keywords:** magnetic bearings, redundant structures, active disturbance rejection controller, anti-interference performance

## Abstract

At present, magnetic bearings are a better energy-saving choice than mechanical bearings in industrial applications. However, there are strongly coupled characteristics in magnetic bearing–rotor systems with redundant structures, and uncertain disturbances in the electrical system as well as external disturbances, and these unfavorable factors degrade the performance of the system. To improve the anti-interference performance of magnetic bearing systems, this paper proposes the inverse of the current distribution matrix *W*^−1^ meaning that the active disturbance rejection control simulation model can be carried out without neglecting the current of each coil. Firstly, based on the working mechanism of magnetic bearings with redundant structures and the nonlinear electromagnetic force model, the current and displacement stiffness models of magnetic bearings are established, and a dynamic model of the rotor is constructed. Then, according to the dynamic model of the rotor and the mapping relationship between the current of each coil and the electromagnetic force of the magnetic bearing, we established the equivalent control loop of the magnetic bearing–rotor system with redundant structures. Finally, on the basis of the active disturbance rejection control (ADRC) strategy, we designed a linear active disturbance rejection controller (LADRC) for magnetic bearings with redundant structures under the condition of no coil failure, and a corresponding simulation was carried out. The results demonstrate that compared to PID+current distribution control strategy, the LADRC+current distribution control strategy proposed in this paper is able to effectively improve the anti-interference performance of the rotors supported by magnetic bearings with redundant structures.

## 1. Introduction

Magnetic bearings have been increasingly being applied in the support units of rotating machinery in recent years, such as in turbo-molecular vacuum pumps, control moment gyroscopes, compressors, and flywheel energy storage systems [1,2,3,4]. Compared with mechanical bearings, magnetic bearings have some superior characteristics including absence of physical contact, low rotation friction, and long life. However, magnetic bearing–rotor systems are complex mechatronics systems, and include the coil, electric power, the controller, the power amplifier, the position sensor, the current sensor, and so on. The failure of any section in the system will make the system unable to continue working, and the rotor will fail. Hence, magnetic bearings with redundant structures for when there is a fault in the coils or power amplifiers were proposed in [5].

Heteropolar magnetic bearings possess features of strongly coupled fluxes and magnetic circuits in adjacent poles. According to these characteristics, Maslen and Meeker et al. proposed magnetic bearings with redundant structures, and then designed the corresponding fault-tolerant control law. The core of the fault-tolerant control law is to design a current distribution strategy for the remaining normal coils and guarantee the linear relationship between the currents and the electromagnetic force. Based on the theory of magnetic bearings with redundant structures, Na and Palazzolo [6] proposed a Lagrange multiplier approach for optimizing the current distribution matrix for conditions of the failure of some coils in a bearing, and established a stiffness model of magnetic bearings with redundant structures. The simulation results verified the effectiveness of their method. Then, to prove the effectiveness of the fault-tolerant controller, Na and Palazzolo [7] carried out experiments on the magnetic bearing–flexible rotor platform, and the experiment results were in good agreement with the system simulation results demonstrated in [6]. M.D. Noh, S-R. Cho, et al. [8] considered faults in the position sensors in magnetic bearing systems, and applied the fault-tolerant magnetic bearing system to a turbo-molecular vacuum pump. The tested rotor remained levitated with the simultaneous failure of three coils while at a rotor speed of 4200 rpm. Additionally, in order to realize the design of a fault-tolerant controller for application in magnetic bearings with an arbitrary number of poles, Meeker [9] proposed an unbiased control strategy to design a fault-tolerant controller. Due to the current distribution matrix reflecting the intrinsic properties of the magnetic bearing, the more physical characteristics of the system included in the process of solving current distribution matrix, the better the performance of the corresponding controller will be. Therefore, in the face of magnetic leakage, eddy current, and the reluctance of the ferromagnetic material path factors of magnetic bearing systems, Na and Palazzolo [10,11,12] introduced a compensation coefficient into the process of solving the current distribution matrix. Considering that the rotor vibrates with a tiny displacement near the equilibrium position, X. Cheng and Baixin Cheng [13] proposed a method that included the rotor at nonequilibrium positions to calculate the current distribution matrix, and the simulation results indicated that the electromagnetic force was closer to the desired electromagnetic force, while the corresponding fault-tolerant controller was tested by simulation [14]. Additionally, in accordance with the requirements of fault-tolerant control systems, Cheng, X. et al. [15] designed fault-tolerant control scheme that consisted of dual DSP microprocessors and a power amplifier. For stators with an even number of evenly spaces poles of equal area, in order to simplify the process for designing fault-tolerant controllers, David Meeker and Eric Maslen [16] proposed an approach that only requires a small number of parameters. On the basis of the aforementioned analysis, it can be concluded that almost all studies have focused on obtaining the current distribution matrix with the aim of optimizing the fault-tolerant controller.

Compared with magnetic bearings with redundant structures, magnetic bearing–rotor systems controlled by differential control strategies have been studied extensively. To solve the problem of rotor vibration caused by mass imbalance, Cui P, He J, et al. [17] proposed a method of identifying the static mass imbalance of the rotor and designed an adaptive null vibration control to suppress the unbalanced vibration. Based on the theory of the least mean squares method and the influence coefficient method, Zhou Jian et al. [18] proposed an online imbalance compensation algorithm, and the results demonstrated that the proposed control algorithm was able to effectively suppress rotor vibration caused by mass imbalance. Additionally, inevitable manufacturing errors in the rotor and the stator introduce harmonic disturbance into the controller, further causing rotor vibration. To address this problem, Borquegallego G, Rossini L and Achtnich T, et al. [19] proposed a novel generalized notch filter in accordance with the character of the disturbance, and compared the results with those of other imbalance control techniques, whereby the proposed control algorithm presented the advantages of not requiring a time-consuming gain matrix or phase shift fine-tuning in order to yield a stable closed-loop system. According to the theory of repetitive control law, Cui et al. [20,21,22,23] proposed a series of repetitive controllers to suppress the harmonic vibration of the magnetic bearing–rotor system. P. Cui [24] and J. He [25] proposed a multiple phase shift notch filter method for magnetic bearing systems to suppress the harmonic components over the full speed range. Moreover, to cope with vibrations caused by parameter uncertainty, external disturbances, or unmodeled disturbances, some robust controllers have been applied to magnetic bearing systems. On the basis of the dynamic characteristics of flexible rotors suspended by magnetic bearings, Ran S. et al. [26] designed a robust H_∞_ controller, and the experimental results suggested that the controller had good performance for suppressing the vibration of the rotor. Jin C., Guo K., Xu Y., et al. [27] treated the disturbance of the system as a total disturbance, and designed a linear active disturbance rejection controller for magnetic bearings. For traditional magnetic bearings, the electromagnetic force of a certain degree of freedom direction and the control current of other degrees of freedom can be considered as a decoupling relationship. For example, the electromagnetic force generated by the corresponding magnetic pole in the *x* direction is only related to the control current $i_{x}$. However, for magnetic bearings with redundant structures, the electromagnetic force in a certain degree of freedom depends on the electromagnetic force produced by all poles of the magnetic bearing, and the electromagnetic force of some poles is not only related to the control current $i_{x}$, but also to $i_{y}$. The coupling characteristics of magnetic bearings with redundant structures causes uncertain disturbances in the system. To improve the anti-interference performance of the system, we introduced a linear active disturbance rejection law into the control system of magnetic bearings with redundant structures. The simulation and experimental results showed that the proposed linear active disturbance rejection controller has better anti-interference performance than the PID controller.

In this paper, under no coil failure conditions in magnetic bearings with redundant structures, in order to improve the anti-interference ability of rotors suspended by magnetic bearings, we established an equivalent rotor dynamic model on the basis of the current and displacement stiffness models of magnetic bearings with redundant structures, and then LADRC was designed and applied to the system. Finally, a simulation was carried out, and results showed that, compared with the PID+current distribution control strategy, the proposed LADRC+current distribution control strategy was able to effectively improve the anti-interference performance of magnetic bearings with redundant structures. The innovation of this paper is that the inverse of the current distribution matrix is proposed and used to construct the relationship between the control current and the currents of coils, so that the relationship between electromagnetic force and each coil current can be converted into the equivalent force mode with the current stiffness and displacement stiffness. Therefore, the LADRC was designed, and the corresponding simulation work was carried out without neglecting the current of each coil $I={\left[\begin{array}{cccc} I_{1} & I_{2} & \ldots & I_{n} \end{array}\right]}^{T}$. By using the method proposed in this paper, we were not only able to use the control logic current to design the LADRC controller, but we could also analyze the current characteristics of the coils using the simulation model.

The paper is organized as follows. The model of magnetic bearings with redundant structures is established and the equivalent control strategy is described in Section 2. The theoretical aspects regarding ADRC are expounded, and LADRC is designed for magnetic bearings with redundant structures in Section 3. The simulation results of the system with the LADRC+current distribution control and the PID+current distribution control are discussed in Section 4. Section 5 provides the conclusions.

## 2. Mathematical Model of Magnetic Bearing with Redundant Structures

**Figure 1:**
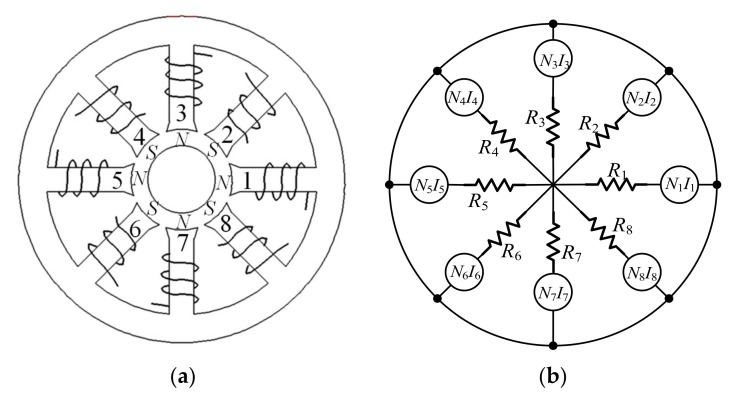
Eight-pole bearing arrangement (**a**) and equivalent magnetic circuit (**b**).

The radial magnetic bearing with redundant structures studied in this paper is shown in Figure 1a, there are eight coils wrapped around a pole independently of each other in the magnetic bearing. According to the relationship between the electromagnetic forces and each coil of the magnetic bearing, the nonlinear electromagnetic forces model is constructed. Based on the idea of the model inverse and the linearization condition of electromagnetic force, the current distribution controller is obtained. In this paper, we take the symmetrical radial magnetic bearing as the research object and design an LADRC+current distribution controller to replace the PID+current distribution controller for magnetic bearings with redundant structures.

### 2.1. Theory of Eight-Pole Magnetic Bearings with Redundant Structures

The equivalent magnetic circuit model of the radial magnetic bearing is shown in Figure 1b. For magnetic bearings with redundant structures, Maslen and Meeker [5] established the relevant equations as shown in Equations (1)–(10).

Considering the complex characteristics of magnetic bearings with redundant structures, the magnetic leakage, eddy current and reluctance of the ferromagnetic material are neglected and the flux density is considered to be uniform while the rotor is at the equilibrium position. Based on the theory of the Ampere Loop, the relationship between currents and magnetic flux can be described as [5]

(1) RΦ=NI

where ***R*** is the reluctance matrix of the magnetic circuit, $\phi_{j}$, ***N***, and ***I*** are the magnetic flux matrix, turns matrix of the coils, and current matrix in the coils, respectively. The reluctance of each magnetic circuit can be calculated as

(2) $$R_{j}=\frac{g_{j}}{\mu_{0}A_{j}}$$

where *g_j_* denotes the air gap between the rotor and *j*th pole, it can be calculated by

(3) $$g_{j}=g_{0}-x\cos\theta_{j}-y\sin\theta_{j}$$

$\theta_{j}$ represents the angle between the *j*th pole and *x* direction.

Assuming that the magnetic flux density is uniform in the gap, the flux matrix can be described as

(4) Φ=AB

then

(5) $$B=A^{-1}R^{-1}NI=VI$$

where ***A*** is the diagonal matrix of the pole area, ***B*** is the magnetic flux density matrix of the air gap.

To satisfy the necessary conditions of the bias current linearization for magnetic bearings with redundant structures, the current distribution matrix ***W*** is introduced into the electromagnetic force model. The essence of the current distribution matrix ***W*** is the structural characteristics of the magnetic bearing, which reflects the relationship between the current of each coil and the logic current. The current distribution matrix can be defined as

(6) $$W=\left[w_{b},w_{x},w_{y}\right]$$

where ***w****_b_* represents the bias current vector, and ***w****_x_* and ***w****_y_* represent the current control vectors in the *x* and *y* directions, respectively. The current matrix will be described as

(7) $$I=W\left[\begin{array}{c} C_{0}\\ i_{x}\\ i_{y} \end{array}\right]=WI_{c}$$

where $C_{0}$ denotes the bias current coefficient and *C*_0_ *=* 4 in this paper, *i_x_*, and *i_y_* represent the logical current of the *x* and *y* directions, respectively. The magnetic forces model [14] can be described as

(8) $$F_{x}(x,y)=I_{c}^{T}W^{T}M_{x}WI_{c}$$

(9) $$F_{y}(x,y)=I_{c}^{T}W^{T}M_{y}WI_{c}$$

where

(10) $$\left\{\begin{array}{c} M_{x}(x,y)=-V^{T}D_{x}V\\ M_{y}(x,y)=-V^{T}D_{y}V\\ D_{x}=\frac{A_{j}}{2\mu_{0}}diag\left[\cos\theta_{j}\right]\\ D_{y}=\frac{A_{j}}{2\mu_{0}}diag\left[\sin\theta_{j}\right] \end{array}\right.$$

However, in order to design a linear controller, the linearization of electromagnetic force needs to be ensured. The current distribution matrix ***W***(*x,y*) must satisfy Equation (11).

(11) $$\left\{\begin{array}{c} W^{T}(x,y)M_{x}W(x,y)-Q_{x}=0\\ W^{T}(x,y)M_{y}W(x,y)-Q_{y}=0 \end{array}\right.$$

where matrices *Q_x_* and *Q_y_* are expressed as

(12) $$Q_{x}=\left[\begin{array}{ccc} 0 & 0.5 & 0\\ 0.5 & 0 & 0\\ 0 & 0 & 0 \end{array}\right],Q_{y}=\left[\begin{array}{ccc} 0 & 0 & 0.5\\ 0 & 0 & 0\\ 0.5 & 0 & 0 \end{array}\right]$$

Finally, the electromagnetic forces can be linearized, and are written as

(13) $$\left\{\begin{array}{c} F_{x}(x,y)=C_{0}i_{x}\\ F_{y}(x,y)=C_{0}i_{y} \end{array}\right.$$

Obviously, the key to the current linearization of redundant magnetic bearings is to find the corresponding current distribution matrix ***W***(*x,y*). In this paper, in order to obtain the current distribution matrix, we adopt a similar method to that proposed in [5].

### 2.2. Linearized Forces with Stiffness Model of Magnetic Bearings with Redundant Structures

A valid current distribution matrix can both linearize and decouple the multiple poles failing due to magnetic bearing forces. The current distribution matrix for some or no coil failure cases can be applied to the controller. While the magnetic suspension rotor is at or near the tiny displacement equilibrium position, the electromagnetic force forms a linear relationship with the displacement and the control currents *i_x_* and *i_y_*. Therefore, according to Equations (9) and (10) and the theory of Taylor series expansion, the linearized resultant forces in the *x* and *y* directions can be expressed as

(14) $$F_{x}(x,y,i_{x},i_{y})\approx \frac{\partial F_{x}}{\partial x}\left|{}_{\begin{array}{l} x=0\\ y=0\\ i_{x}=0\\ i_{y}=0 \end{array}}\right.x+\frac{\partial F_{x}}{\partial y}\left|{}_{\begin{array}{l} x=0\\ y=0\\ i_{x}=0\\ i_{y}=0 \end{array}}\right.y+\frac{\partial F_{x}}{\partial i_{x}}\left|{}_{\begin{array}{l} x=0\\ y=0\\ i_{x}=0\\ i_{y}=0 \end{array}}\right.i_{x}+\frac{\partial F_{x}}{\partial i_{y}}\left|{}_{\begin{array}{l} x=0\\ y=0\\ i_{x}=0\\ i_{y}=0 \end{array}}\right.i_{y}$$

(15) $$F_{y}(x,y,i_{x},i_{y})\approx \frac{\partial F_{y}}{\partial x}\left|{}_{\begin{array}{l} x=0\\ y=0\\ i_{x}=0\\ i_{y}=0 \end{array}}\right.x+\frac{\partial F_{y}}{\partial y}\left|{}_{\begin{array}{l} x=0\\ y=0\\ i_{x}=0\\ i_{y}=0 \end{array}}\right.y+\frac{\partial F_{y}}{\partial i_{x}}\left|{}_{\begin{array}{l} x=0\\ y=0\\ i_{x}=0\\ i_{y}=0 \end{array}}\right.i_{x}+\frac{\partial F_{y}}{\partial i_{y}}\left|{}_{\begin{array}{l} x=0\\ y=0\\ i_{x}=0\\ i_{y}=0 \end{array}}\right.i_{y}$$

According to the model of magnetic bearings presented in Equations (11) and (12), and the definition of current distribution matrix ***W***, we calculate the partial derivatives of $F_{x}$ and $F_{y}$ with respect to *x*, *y*, *i_x_* and *i_y_*, and the corresponding equations are described as Equations (16)–(23).

Furthermore, Equations (16)–(23) also represent the position stiffness and current stiffness. Therefore, we define the position stiffness as $K_{xx}$,$K_{xy}$,$K_{yx}$ and $K_{yy}$, and current stiffness is $K_{i}{}_{{}_{xx}}$,$K_{i}{}_{{}_{xy}}$,$K_{i}{}_{{}_{yx}}$ and $K_{i}{}_{{}_{yy}}$. The corresponding mathematical formulas for position stiffness are shown as follows

(16) $$-K_{xx}=\frac{\partial F_{x}}{\partial x}\left|{}_{\begin{array}{l} x=0\\ y=0\\ i_{x}=0\\ i_{y}=0 \end{array}}\right.=w_{b}^{T}\frac{\partial M_{x}}{\partial x}w_{b}C_{0}^{2}\left|{}_{\begin{array}{l} x=0\\ y=0 \end{array}}\right.=w_{b}^{T}M_{xx0}w_{b}C_{0}^{2}$$

(17) $$-K_{xy}=\frac{\partial F_{x}}{\partial y}\left|{}_{\begin{array}{l} x=0\\ y=0\\ i_{x}=0\\ i_{y}=0 \end{array}}\right.=w_{b}^{T}\frac{\partial M_{x}}{\partial y}w_{b}C_{0}^{2}\left|{}_{\begin{array}{l} x=0\\ y=0 \end{array}}\right.=w_{b}^{T}M_{xy0}w_{b}C_{0}^{2}$$

(18) $$-K_{yx}=\frac{\partial F_{y}}{\partial x}\left|{}_{\begin{array}{l} x=0\\ y=0\\ i_{x}=0\\ i_{y}=0 \end{array}}\right.=w_{b}^{T}\frac{\partial M_{y}}{\partial x}w_{b}C_{0}^{2}\left|{}_{\begin{array}{l} x=0\\ y=0 \end{array}}\right.=w_{b}^{T}M_{yx0}w_{b}C_{0}^{2}$$

(19) $$-K_{yy}=\frac{\partial F_{y}}{\partial y}\left|{}_{\begin{array}{l} x=0\\ y=0\\ i_{x}=0\\ i_{y}=0 \end{array}}\right.=w_{b}^{T}\frac{\partial M_{y}}{\partial y}w_{b}C_{0}^{2}\left|{}_{\begin{array}{l} x=0\\ y=0 \end{array}}\right.=w_{b}^{T}M_{yy0}w_{b}C_{0}^{2}$$

and the current stiffness can be calculated as

(20) $$K_{i}{}_{{}_{xx}}=\frac{\partial F_{x}}{\partial I_{c}}\frac{\partial I_{c}}{\partial i_{x}}\left|{}_{\begin{array}{l} x=0\\ y=0\\ i_{x}=0\\ i_{y}=0 \end{array}}\right.=w_{x}^{T}M_{x}w_{b}C_{0}\left|{}_{\begin{array}{l} x=0\\ y=0 \end{array}}\right.+w_{b}^{T}M_{x}w_{x}C_{0}\left|{}_{\begin{array}{l} x=0\\ y=0 \end{array}}\right.$$

(21) $$K_{i}{}_{{}_{xy}}=\frac{\partial F_{x}}{\partial I_{c}}\frac{\partial I_{c}}{\partial i_{y}}\left|{}_{\begin{array}{l} x=0\\ y=0\\ i_{x}=0\\ i_{y}=0 \end{array}}\right.=w_{y}^{T}M_{x}w_{b}C_{0}\left|{}_{\begin{array}{l} x=0\\ y=0 \end{array}}\right.+w_{b}^{T}M_{x}w_{y}C_{0}\left|{}_{\begin{array}{l} x=0\\ y=0 \end{array}}\right.$$

(22) $$K_{i}{}_{{}_{yx}}=\frac{\partial F_{x}}{\partial I_{c}}\frac{\partial I_{c}}{\partial i_{y}}\left|{}_{\begin{array}{l} x=0\\ y=0\\ i_{x}=0\\ i_{y}=0 \end{array}}\right.=w_{x}^{T}M_{y}w_{b}C_{0}\left|{}_{\begin{array}{l} x=0\\ y=0 \end{array}}\right.+w_{b}^{T}M_{y}w_{x}C_{0}\left|{}_{\begin{array}{l} x=0\\ y=0 \end{array}}\right.$$

(23) $$K_{i}{}_{{}_{yy}}=\frac{\partial F_{x}}{\partial I_{c}}\frac{\partial I_{c}}{\partial i_{y}}\left|{}_{\begin{array}{l} x=0\\ y=0\\ i_{x}=0\\ i_{y}=0 \end{array}}\right.=w_{y}^{T}M_{y}w_{b}C_{0}\left|{}_{\begin{array}{l} x=0\\ y=0 \end{array}}\right.+w_{b}^{T}M_{y}w_{y}C_{0}\left|{}_{\begin{array}{l} x=0\\ y=0 \end{array}}\right.$$

According to the above analysis, while the rotor is located at or near the equilibrium position, the magnetic forces can be considered to be linear. Hence, the magnetic force model with the position stiffness and current stiffness will be established. By this method, a control law that is based on the model of the system can be designed to improve the stability of the system.

For the magnetic bearing and rotor studied in this paper, the structural parameters are shown in Table 1.

While there is no coil failure in the magnetic bearing, the current distribution matrix ***W*** can be expressed as [5]

(24) $$W=\frac{g_{0}}{4N\sqrt{\mu_{0}A}}\left[\begin{array}{ccc} 2 & 2 & 0\\ -2 & -\sqrt{2} & -\sqrt{2}\\ 2 & 0 & 2\\ -2 & \sqrt{2} & -\sqrt{2}\\ 2 & -2 & 0\\ -2 & \sqrt{2} & \sqrt{2}\\ 2 & 0 & -2\\ -2 & -\sqrt{2} & \sqrt{2} \end{array}\right]$$

Based on the structural parameters of the magnetic bearing and Equation (24), the values of stiffness can be obtained, and these are *K_xx_* = −40,000 N/m, *K_xy_* = *K_yx_* = 0, *K_yy_* = −40,000 N/m; *K_ix_* = 4 A/m, *K_ixy_* = *K_iyx_* = 0, *K_iyy_* = 4 A/m. Therefore, the electromagnetic forces of eight-pole symmetrical radial magnetic bearing studied in this paper can also be described as

(25) $$\begin{array}{l} F_{x}=-K_{xx}x+K_{i}{}_{{}_{xx}}i_{x}\\ F_{y}=-K_{yy}y+K_{i}{}_{{}_{yy}}i_{y} \end{array}$$

Additionally, considering the disturbance and neglecting the torque of the rotor, the rotor dynamic equation in *x* and *y* directions can be expressed as follows

(26) $$\begin{array}{l} m\ddot{x}=F_{x}+F_{ex}\\ m\ddot{y}=F_{y}+F_{ey} \end{array}$$

We select the displacement x and velocity $\dot{x}$ of the rotor as the state variable. The control logic current $i_{x}$ is the input *u* (*t*) of the rotor system, y(t) is the output of the rotor system. Considering the disturbance force $F_{ex}$, and defining $x_{1}=x$ and $x_{2}=\dot{x}$, the state space equation of the system is written as

(27) $$\left\{\begin{array}{l} {\dot{x}}_{1}=x_{2}\\ {\dot{x}}_{2}=-\frac{k_{xx}}{m}x_{1}+\frac{k_{ixx}}{m}u(t)+\frac{F_{ex}}{m}\\ y(t)=x_{1} \end{array}\right.$$

### 2.3. Equivalent Control Strategy of Magnetic Bearings with Redundant Structures

**Figure 2:**
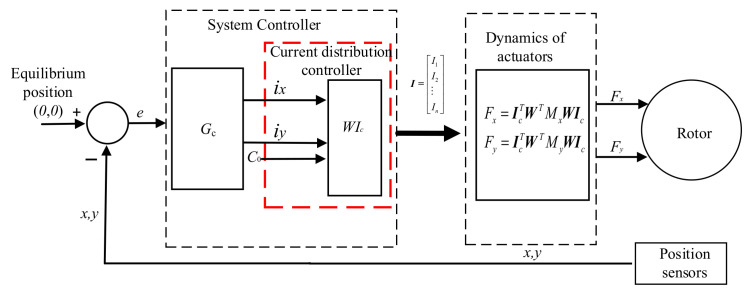
Closed-loop control strategy in magnetic bearings with redundant structures.

Generally, for magnetic bearings with redundant structures, the system controller needs to meet two requirements. One is that there must be a position control law *G*_c_, and the other is the requirement for a current distribution controller. Because the current distribution matrix ***W*** reflects the relationship between the control logic currents and the current of each coil, ***W*** is usually designed as a current distribution controller. Therefore, the entire system, including controllers, can be described in Figure 2.

As shown in Figure 2, according to the logic control currents *i_x_*, *i_y_*, and the bias current coefficient, the current distribution controller generates the currents of coils. Then, the actuators generate electromagnetic forces to ensure the rotor remains in suspension. In the current related research work, the position control law *G*_c_ usually adopts the PID control algorithm, which does not depend on the model of the controlled object. However, some advanced control strategies have been designed using the model of the controlled object. To use the linearized electromagnetic force, as shown in Equation (25), to design an advanced controller, the relationship between the logic current and the current of each coil was deeply analyzed. As depicted in Figure 2, we can assume that the output of the position control corresponds to the desired logic control currents i*_x_ and i*_y_, and the current distribution controller produces the currents and then the magnetic bearing will generate the virtual logic control currents *i_x_* and *i_y_* in the system. Therefore, we adopt the inverse of the current distribution matrix and the current of each coil to form logic control currents, and the relationship can be defined as

(28) $$I_{c}=W^{-1}I$$

**Figure 3:**
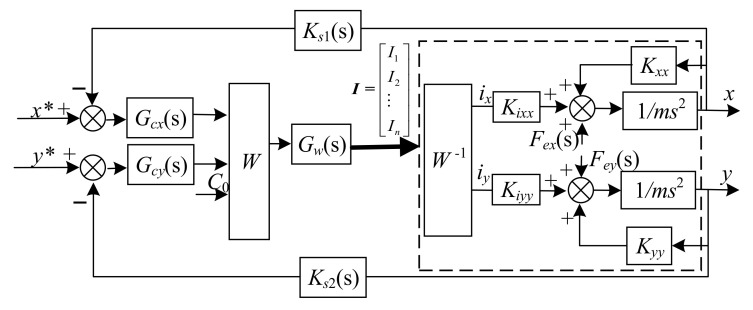
Closed-loop equivalent control schematic diagram of the entire system.

On the basis of the mathematical relationship in Equation (28), the equivalent control schematic diagram of the entire system can be depicted as in Figure 3.

**Figure 4:**
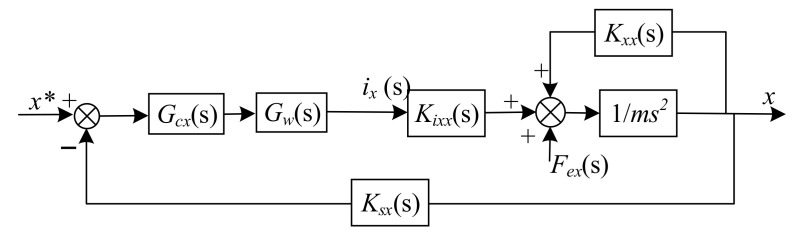
Equivalent control schematic diagram of *x* direction.

In Figure 3, the x*, y*, i*_x_, and i*_y_ represent the desired position and logic control currents respectively. *G_cx_* and *G_cy_* are the position control laws of the *x* and *y* directions; *G_w_* is the transfer function of the amplifier, and *G_w_* = 1 A/V; *K_sx_* and *K_sy_* are the gain of displacement sensors. *I*(s) is a matrix that denotes the currents of each coil. From Figure 3, although the currents of the coils determine the displacement of the rotor in both *x* and *y* directions, by using the model of linearized resultant forces, the motion of the rotor can be considered decoupled in *x* and *y* directions. Hence, the equivalent control schematic diagram of the rotor in the *x* direction is described in Figure 4.

From Figure 3 or Figure 4, it can be seen that there is a disturbance $F_{ex}$ in the *x* direction control loop, and the same thing occurs in the *y* direction control loop. These disturbances will reduce the stability of the system. To suppress the disturbance $F_{ex}$, according to the equivalent control loop of *x* direction is described in Figure 4, we adopt the linear active disturbance rejection (LADRC) algorithm as the position control law, and the same for the *y* direction.

## 3. Design of ADRC for AMB with Redundant Structures

### 3.1. Basic Theory and Structure of ADRC

Active disturbance rejection controllers consist of a tracking differentiator (TD), the state feedback error control law, the extended state observer (ESO), and a compensation device for disturbance estimation. In ADRC theory, the system parameter perturbation, the model perturbation, and the external disturbance are taken as the total disturbance of system. The ESO makes use of the input and output of the object to estimate the total disturbance of the real-time system, and the total disturbance is eliminated by the compensation device. The core function of ADRC is that it can estimate and compensate the disturbance in real time. Additionally, the function of TD will produce *n*th-differential values of the desired signal $x^{*}$, which are $x_{1},\;x_{2}\cdots x_{n}$. Then, the feedback error controller will use the errors between $x_{1},\;x_{2}\cdots x_{n}$ and ${\hat{x}}_{1},\;{\hat{x}}_{2}\cdots {\hat{x}}_{n}$ to control the whole system.

**Figure 5:**
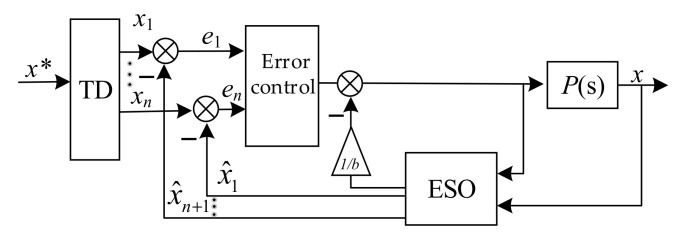
System structure of ADRC.

For an *n*th-order system *P*(*s*) that includes disturbance, *f* can be expressed as shown in Equation (29), while the basic structure of ADRC is shown in Figure 5.

(29) $$\left\{\begin{array}{l} {\dot{x}}_{1}=x_{2}\\ {\dot{x}}_{2}=x_{3}\\ \vdots \\ {\dot{x}}_{n-1}=x_{n}\\ \begin{array}{l} {\dot{x}}_{n}=f(x,\dot{x}\ldots x^{\left(n-1\right)})+bu+f\\ y=x_{1} \end{array} \end{array}\right.$$

Figure 5 demonstrates that without the internal structure of the object, the ESO can estimate the state variables of the system by the input and output of the controlled object *P*(s). The system disturbance is defined as an expansion state variable that can be estimated by ESO. While the state observer satisfies the stability condition, the estimate variables will track each state of the system as well as the total disturbances, that is, ${\hat{x}}_{i}\to x_{i}$ and ${\hat{x}}_{n+1}\to f(x,\dot{x}\ldots x^{\left(n-1\right)},\omega (t))$. Considering that the given signal $x^{*}$ of the magnetic bearing–rotor system is a constant, TD is not adopted in this paper. For magnetic bearings with redundant structures, the coupling effects of poles, the imbalance force of the rotor, and coupling effects of the rotor in different degrees of freedom can be considered as the disturbance ω(t).

For the system described in Equation (29), the corresponding expansion state equation can be designed as follows

(30) $$\left\{\begin{array}{l} {\dot{x}}_{1}=x_{2}\\ {\dot{x}}_{2}=x_{3}\\ \vdots \\ {\dot{x}}_{n-1}=x_{n}\\ {\dot{x}}_{n}=f(x,\dot{x}\ldots x^{\left(n-1\right)})+bu\\ {\dot{x}}_{n+1}=\omega (t)\\ y=x_{1} \end{array}\right.$$

The LESO of Equation (30) is defined as

(31) $$\left\{\begin{array}{l} e_{1}={\hat{x}}_{1}-x_{1}\\ \begin{array}{l} {\dot{\hat{x}}}_{1}={\hat{x}}_{2}-\beta_{01}e_{1}\\ {\dot{\hat{x}}}_{2}={\hat{x}}_{3}-\beta_{02}e_{1} \end{array}\\ \vdots \\ {\dot{\hat{x}}}_{n}={\hat{x}}_{n+1}+bu-\beta_{n}e_{1}\\ {\dot{\hat{x}}}_{n+1}=-\beta_{n+1}e_{1}\\ \hat{y}={\hat{x}}_{1} \end{array}\right.$$

where the $\beta_{01}$,$\beta_{02}$…$\beta_{0n}$ are the gains of the LESO and these parameters determine the performance of LESO, $e_{1}$ is the error of between estimate and actual value of $x_{1}$.

According to the real-time estimate of the LESO, the disturbance information will be compensated in the system. By introducing the new input $u_{0}(t)$, the value of the control after compensation is defined as

(32) $$u(t)=u_{0}(t)-z_{n+1}/b$$

By taking the control amount u(t) into the original system shown in Equation (29), Equation (29) can be transformed into

(33) $$\left\{\begin{array}{l} {\dot{x}}_{1}=x_{2}\\ {\dot{x}}_{2}=x_{3}\\ \vdots \\ {\dot{x}}_{n-1}=x_{n}\\ \begin{array}{l} {\dot{x}}_{n}=f(x,\dot{x}\ldots x^{\left(n-1\right)})+bu_{0}\\ y=x_{1} \end{array} \end{array}\right.$$

It is obvious that the disturbance factor has been eliminated after compensation, and thus the system performance will be improved.

### 3.2. Design of the LADRC for the AMB with Redundant Structures

A typical characteristic of magnetic bearings with redundant structures is that the currents of all of the coils simultaneously determine the electromagnetic forces of the rotor in the *x* and *y* directions, and any disturbance in the control loop of the *x* and *y* directions will affect the stability of the magnetic suspension rotor. However, the equivalent control model of the suspended rotor in the *x* direction degree of freedom has been established as shown in Figure 3, and this is the same as the rotor in the *y* direction. Therefore, in this paper, we design the ADRC according to the rotor in the *x* direction control loop, and then apply the active disturbance rejection controllers to the closed-loop path of the *x* and *y* directions, respectively.

(1) LESO model.

ESO is the most important part of the ADRC. For magnetic bearings with redundant structures, the ESO just requires the system output the displacement of rotor x and the controller output the logic current $i_{x}$, and it can effectively estimate the total disturbance of the system. To reduce the parameters that need to be adjusted, and simplify the controller structure, this paper adopts the LESO described in Equation (31). Compared with the ESO, LESO not only has good estimation performance for the disturbance of magnetic bearings, but the controller also contains four parameters that need to be adjusted.

Based on the dynamic model of the rotor and the LESO model *t*, the corresponding LESO for the magnetic bearing–rotor system can be designed. We expand the disturbance term $F_{ex}/m$ into the new state variable $x_{3}$ and its derivative ${\dot{x}}_{3}=\omega (t)$; the expanded state space equation for magnetic bearings with redundant structures can be obtained as follows

(34) $$\left\{\begin{array}{l} {\dot{x}}_{1}=x_{2}\\ {\dot{x}}_{2}=-\frac{k_{xx}}{m}x_{1}+\frac{k_{ixx}}{m}u(t)+x_{3}\\ {\dot{x}}_{3}=\omega (t)\\ y(t)=x_{1} \end{array}\right.$$

According to Equation (31), the proposed LESO in this paper can be described as

(35) $$\left\{\begin{array}{l} e_{1}={\hat{x}}_{1}-x_{1}\\ {\dot{\hat{x}}}_{1}={\hat{x}}_{2}-\beta_{01}e_{1}\\ {\dot{\hat{x}}}_{2}={\hat{x}}_{3}-\beta_{02}e_{1}-\frac{k_{xx}}{m}{\hat{x}}_{1}+\frac{k_{ixx}}{m}u(t)\\ {\dot{\hat{x}}}_{3}=-\beta_{03}e_{1} \end{array}\right.$$

where $\beta_{01}$$\beta_{02}$$\beta_{03}$ are the gain parameters of the LESO, and u(t) is the control logic current. Suppose the parameter $k_{xx}/m$ is known, and $k_{xx}{\hat{x}}_{1}/m$ denotes the displacement acceleration estimation value of the rotor that contains the displacement stiffness factor. The term $k_{xx}{\hat{x}}_{1}/m$ belongs to the inherent characteristic of the system. Considering that the inherent characteristics of the LESO system can reduce the workload of ${\hat{x}}_{3}$, the LESO is able to estimate the unmolded factors in magnetic bearings with redundant structures.

Additionally, in order to guarantee the estimated performance of LESO, the error between Equation (35) and Equation (34) should be considered. While neglecting the disturbance term of the system model, the error state equation can be obtained as

(36) $$\dot{e}=\left[\begin{array}{c} {\dot{e}}_{1}\\ {\dot{e}}_{2}\\ {\dot{e}}_{3} \end{array}\right]=He=\left[\begin{array}{ccc} -\beta_{01} & 1 & 0\\ -\frac{k_{xx}}{m}-\beta_{02} & 0 & 1\\ -\beta_{03} & 0 & 0 \end{array}\right]\left[\begin{array}{c} e_{1}\\ e_{2}\\ e_{3} \end{array}\right]$$

where e denotes the errors matrix between estimated variables and original variables. To ensure that the observed variables track the actual values, the ***H*** should satisfy the Hurwitz condition.

(2) Design of disturbance compensation.

Based on the analysis of the previous sections, we can design the compensation control law on the basis of the estimated disturbance; according to the model in (32), the compensation control law is obtained as

(37) $$u(t)=u_{0}(t)-{\hat{x}}_{3}/b\;(b=k_{xx}/m)$$

Bringing Equation (37) into Equation (28), the state space equation of the magnetic bearing–rotor system can be changed as follows.

(38) $$\left\{\begin{array}{l} {\dot{x}}_{1}=x_{2}\\ {\dot{x}}_{2}=-\frac{k_{xx}}{m}x_{1}+\frac{k_{ixx}}{m}u_{0}(t)\\ y(t)=x_{1} \end{array}\right.$$

From Equation (38), it can be concluded that while the estimated disturbance ${\hat{x}}_{3}$ can effective track disturbance $F_{ex}/m$, the new input can be introduced as shown Equation (37), meaning that the disturbance term of Equation (27) can be eliminated, and the input $u_{0}(t)$ is the position control law of the system. Therefore, the anti-interference ability of magnetic bearings with redundant structures will be improved.

(3) Design of the control law of feedback error.

Although the disturbance can be eliminated by means of compensation control, the feedback error control law needs to be designed to ensure that the actual displacement of the rotor follows the desired displacement signal, that is $x\to x^{*}$. Considering the PID controller has a simple structure and is widely used in industry, we adopt the PID controller as the control law for feedback error. The transfer function of PID is expressed as

(39) $$G_{cx}(s)=k_{p}+\frac{k_{i}}{s}+k_{d}s$$

Additionally, it is convenient for us to compare the performance of PID with the ADRC controller.

**Figure 6:**
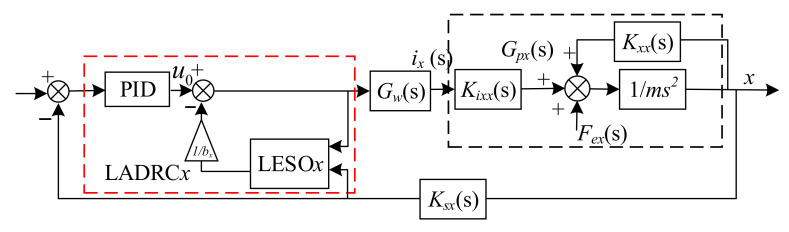
Control schematic diagram of *x* direction with ADRC.

So far, based on the equivalent linearized dynamics model described in Figure 4, we have designed the LADRC for magnetic bearings with redundant structures in the closed-loop control of the *x* direction. The corresponding schematic diagram of the control is shown in Figure 6.

## 4. Simulation and Analysis

**Figure 7:**
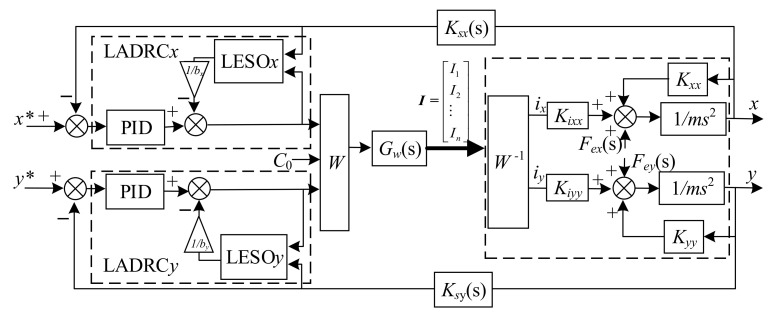
Control block diagram of the entire system with LADRC.

To verify the effectiveness of the proposed control strategy, simulations on an AMB rotor system with redundant structures were carried out using MATLAB/Simulink. The LADRC*x* and LADRC*y* were applied on the closed-loop path of the *x* and *y* directions, respectively. Due to the symmetrical structure of the magnetic bearing, the structure and parameters of LADRC*y* are the same as those of LADRC*x*. Therefore, according to the control strategy depicted in Figure 3, we established the simulation model of the entire system, which is shown in Figure 7.

For the model of LESO, Zheng and Gao [28] proposed a theory that the parameters $\beta_{01}$$\beta_{02}$$\beta_{03}$ could be determined according to the bandwidth $\omega_{0}$ of the linear extended state observer, and this method was adopted in this paper. From Equation (36), we can obtain the characteristic equation as follows

(40) $$\lambda (s)=s^{3}+\beta_{01}s^{2}+(\beta_{02}-k_{xx}x/m)s+\beta_{03}$$

By using the characteristic equation ${\left(s+\omega_{0}\right)}^{3}$, the third polar poles of LESO can be configured $-\omega_{0}$. Hence, we can get

(41) $$\begin{array}{ll} {\left(s+\omega_{0}\right)}^{3} & =s^{3}+3\omega_{0}s^{2}+3\omega_{0}^{2}s+\omega_{0}^{3}\\ & =s^{3}+\beta_{01}s^{2}+(\beta_{02}-k_{xx}x/m)s+\beta_{03} \end{array}$$

Then, the gain parameters of LESO $\beta_{01}$$\beta_{02}$$\beta_{03}$ are equivalent to $3\omega_{0}$,$3\omega_{0}^{2}+k_{xx}x/m$,$\omega_{0}^{3}$. In this paper, we define $\omega_{0}=3000$, and it is also guaranteed that matrix ***H*** satisfies the Hurwitz condition. In particular, it is stated that the parameters of the LADRC*y* are consistent with the relevant parameters in the LADRC*x*. In Figure 7, *b_x_* and *b_y_* represent the compensation coefficients of the *x* and *y* directions, respectively, and they are defined as having the same value. According to the mathematical model of the system shown in Equation (37), *b_x_* = *b_y_* = *k_xx_*/m. The parameters of PID*x* and PID*y* are defined as *k_px_* = *k_py_* = 20, *k_ix_* = *k_iy_* = 2, *k_dx_* = *k_dy_* = 0.02.

Considering that the periodic disturbance is a typical type of disturbance for the magnetic bearing–rotor system, and in order to verify the disturbance rejection capability of the LADRC, the periodic step and sinusoidal disturbance are considered in this paper. Additionally, the estimated performance of LESO is very important for the LADRC. The accurate estimation of LSEO can enable LADRC to effectively compensate the disturbance of the system, improving the disturbance suppression ability of the system. Therefore, in the simulation section, we also evaluate the estimation performance of LESO*x*.

(1) Test with periodic step disturbance.

**Figure 8:**
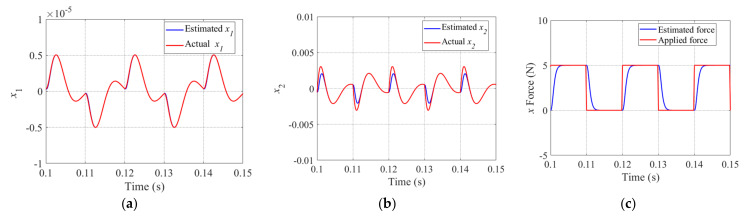
Actual variable (red line) and estimated variable (blue line) under periodic step disturbance for $x_{1}$ (**a**), $x_{2}$ (**b**), and disturbance force *F_ex_* (**c**).

For rotors suspended under magnetic bearings with redundant structures, we define the periodic step disturbance forces as *F_ex_* and *F_ey_*, and they are applied to the *x* and *y* directions of the rotor. The corresponding amplitudes of *F_ex_* and *F_ey_* are 5 and −5, and they have the same frequency of 50 Hz. As described in Equation (35), the LESO*x* can estimate the state variables $x_{1}$,$x_{2}$ and $x_{3}$, which represent the displacement, velocity, and acceleration of the rotor, respectively. The corresponding tracking performance of LESO is shown in Figure 8, and in order to conveniently evaluate the estimation effect of the interference force, we convert $x_{3}$ to the expression of disturbance force. Additionally, as shown in Figure 8, the red and blue lines denote the actual and estimated values, respectively. It is obvious that the LESO*x* is able to effectively estimate the displacement, velocity and disturbance of the rotor, and the estimated force will be used for the compensation control law in order to eliminate the disturbance term from the system.

For the periodic step disturbance applied to the *x* direction of the rotor, Figure 8a denotes the estimated displacement value by completely tracking the actual value. Because there are periodic changes in the applied force, it is inevitable that there will be some errors between the estimated and the actual values of velocity and disturbance force, and these are shown in Figure 8b,c respectively. However, the LESO*x* was able to estimate most of the values of the corresponding state variables in the system, especially for the disturbance force.

**Figure 9:**
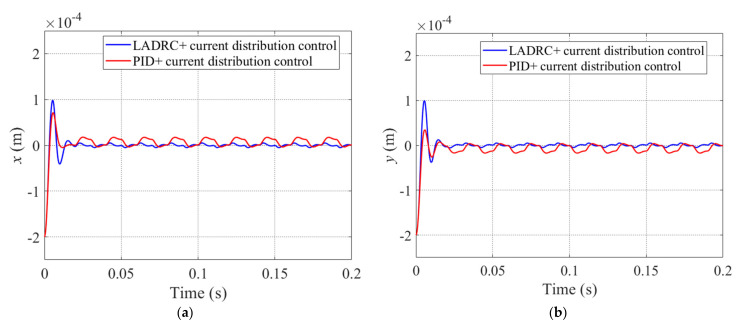
Trajectories of rotor in *x* direction (**a**) and *y* direction (**b**) under periodic step disturbance for PID+current distribution control (red line) and LADRC+current distribution control (blue line).

The displacements of the rotor in *x* and *y* directions while the periodic step disturbance forces *F_ex_* and *F_ey_* are acting on the system are shown in Figure 9a,b. The red line denotes the trajectory of the rotor under the PID+current distribution control, and the blue line denotes the trajectory of the rotor under the LADRC+current distribution control. As shown in Figure 9a, while the rotor is in the stable equilibrium state and under PID+current distribution control strategy, the maximum displacement fluctuation value of the rotor which is in the *x* direction is about 0.3 × 10^−4^ m and it needs about 0.018 s to return to the equilibrium position. However, while the system adopts the LADRC+current distribution control strategy, the corresponding value of the rotor is about 0.1 × 10^−4^ m, and it requires a very short time of about 0.01 s for the rotor to return to equilibrium position. It is obvious that, compared to when the PID+current distribution control strategy is applied to the system, the maximum displacement fluctuation value of the rotor decreased by about 66.7% when using the control strategy proposed in this paper. Additionally, the same thing happens in the *y* direction of the rotor under the corresponding control.

(2) Test under sinusoidal disturbance.

**Figure 10:**
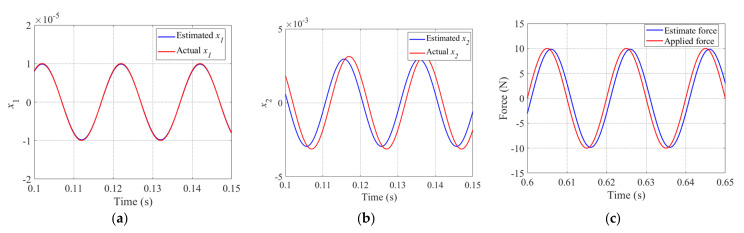
Actual variable (red line) and estimated variable (blue line) under sinusoidal disturbance for $x_{1}$ (**a**), $x_{2}$ (**b**), and disturbance force *F_ex_* (**c**).

In this paper, we adopt sinusoidal disturbance forces described as $F_{ex}=10\sin(2\pi 50t)$ N and $F_{ey}=10\cos(2\pi 50t)$ N, which are applied to the *x* and *y* directions of the rotor. The performance of LESO*x* was tested while sinusoidal disturbance was applied to the system. The actual and estimated state variables $x_{1}$,$x_{2}$ and $x_{3}$ are shown in Figure 10a–c with red lines and blue lines, respectively. Similarly, Figure 10c shows the disturbance force converted from $x_{3}$. It can be seen that the estimated value of *x*_1_ (displacement of the rotor) was tracked by the actual $x_{1}$. Although there is a certain deviation and lag between the estimated and actual value occurring in $x_{2}$ and disturbance force, LESO*x* was still more accurate at estimating the *x*_2_ and the disturbance force. Figure 10 shows that the LESO*x* has a good estimation performance, thus improving the disturbance compensation capability of LADRC*x*.

**Figure 11:**
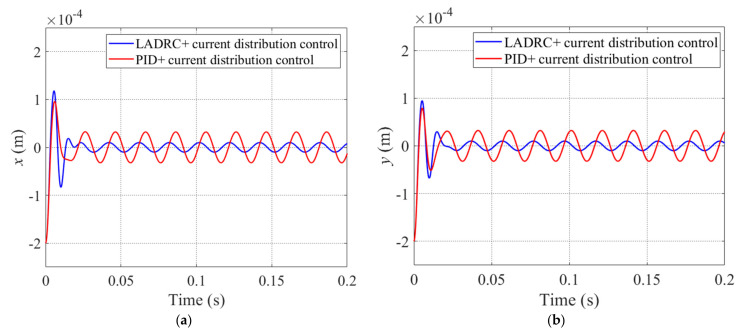
Trajectories of rotor in the *x* direction (**a**) and the *y* direction (**b**) under sinusoidal disturbance for PID+current distribution control (red line) and LADRC+current distribution control (blue line).

Figure 11 shows the displacement waveform of the rotor while sinusoidal disturbance is acting on the system. By analyzing Figure 11a,b, we can draw some conclusions that the maximum displacement fluctuation of the rotor under PID+current distribution control in the *x* direction is about ±0.4 × 10^−4^ m, and while fluctuation in the *y* direction is ±0.4 × 10^−4^ m. However, for the system that adopted the LADRC+current distribution control strategy, the value of *x* fluctuates within ±0.15 × 10^−4^ m, and *y* fluctuates within ±0.1 × 10^−4^ m. It is obvious that the corresponding amplitudes of *x* and *y* decreased by about 62.5% and 75% when the control strategy proposed in this paper was adopted. The results show that, compared with the PID+current distribution control strategy, the proposed LADRC+current distribution control strategy had a good effect on suppressing sinusoidal disturbance.

**Figure 12:**
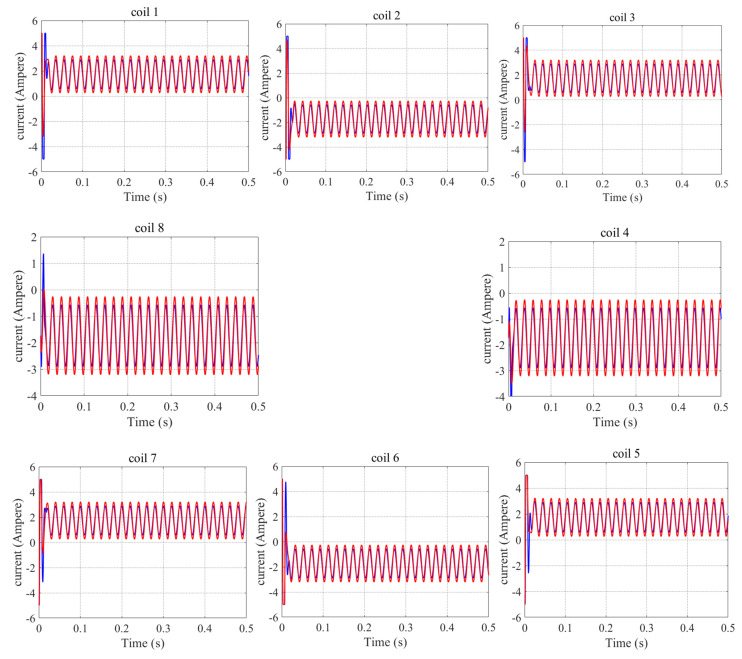
Current response curves of coils (coil 1 coil 2…coil 8) under sinusoidal disturbance for PID+current distribution control (red line) and LADRC+current distribution control (blue line).

The current response curves of the coils are shown in Figure 12 (coil 1 coil 2…coil 8), and the red lines indicate the current response of all coils under the PID+current distribution control strategy, while the blue lines denote the current response of the coils under the LADRC+current distribution control strategy. From Figure 12, we can draw the conclusion that the direction of the current in each coil reflects the characteristics of the poles for heteropolar magnetic bearings. As demonstrated in Figure 12, due to the fact that LADRC suppresses the disturbance of the system, and that system stability is improved, compared with the PID+current distribution control, the value of the current in each coil is decreased when using the LADRC+current distribution control strategy.

## 5. Conclusions

Considering the working principle of magnetic bearings with redundant structures, the stiffness properties and the equivalent closed-loop model are analyzed and established. To suppress the complex disturbances of rotors suspended by magnetic bearings with redundant structures, a linear active disturbance rejection controller was designed for the system. The corresponding simulation was carried out under periodic step disturbance or sinusoidal disturbance, and the results were compared with the PID+current distribution control system. The simulation results demonstrate that:

(1) While the periodic step disturbance or sinusoidal disturbance is acting on the system, the linear extended state observers LESO*x* and LESO*y*, designed for the *x* and *y* directions, respectively, demonstrated good estimation and tracking performance; in particular, the LESO was able to estimate most of the disturbance values in real time. It provides an effective value of disturbance for the compensator of the LADRC, thus ensuring the performance of the LADRC.
(2) For the periodic step disturbance or sinusoidal disturbance of the magnetic bearing–rotor system, compared with the PID+current distribution control, while the rotor is suspended, the maximum vibration displacement of the rotor was attenuated by 70%. The rotor suspension accuracy was greatly improved by using LADRC+current distribution control.

Consequently, for magnetic bearings with redundant structures, compared to the PID+current distribution control strategy, we believe that the proposed LADRC+current distribution control strategy can improve the anti-interference performance of magnetically levitated rotors. We have to admit that the LADRC designed in this paper does not effectively suppress all disturbances. For magnetic bearings with redundant structures, there are strong coupling characteristics in the system, and the disturbances with different properties, such as the different magnitude or frequency of disturbance need to be considered. Therefore, in order to obtain more accurate system parameters, and to ensure that the system has good anti-interference ability under different disturbances, the system identification approach will be adopted and control laws that are based on the characteristics of the disturbance will be studied in future work.

## Figures and Tables

**Table 1:** Structural parameters of magnetic bearing.

Structure Parameter	Value	Unit
Pole area, A0	5.4 × 10^−5^	m^2^
Turns per coil, N	56	/
Pole initial gap, g0	4 × 10^−4^	m
Pole angle, θj	(j−1)π/4	rad
Saturation magnetic-flux density, Bsat	1.2	T
Rotor weight, m	0.8	kg

## Data Availability

Not applicable.
